# International Differences in Outpatient Pain Management: A Survey of Sickle Cell Disease

**DOI:** 10.3390/jcm8122136

**Published:** 2019-12-03

**Authors:** Nadirah El-Amin, Paul Nietert, Julie Kanter

**Affiliations:** 1 Department of Pediatric Hematology-Oncology, Virginia Commonwealth University, Richmond, VA 23219, USA; nadirah.elamin@vcuhealth.org; 2 Department of Public Health Sciences, Medical University of South Carolina, Charleston, SC 29425, USA; nietertpj@musc.edu; 3 Department of Medicine, University of Alabama, Birmingham, AL 35233, USA

**Keywords:** sickle cell, opioid, chronic pain

## Abstract

Vaso-occlusive pain crises are the hallmark of sickle cell disease (SCD) and the primary reason for health care utilization. Both national and international guidelines recommend aggressive intravenous opioids, intravenous fluids and anti-inflammatory therapy as the mainstay of treatment for acute SCD pain. However, many vaso-occlusive crises are managed at home with oral medication and supportive care. There are no guidelines on home medication management of SCD-related pain, likely due to the lack of well-defined endpoints for acute events and the lack of funding for already approved pain medications. Amplifying this issue is the growing concern for opioid abuse and misuse in the United States (US) and internationally. This study aimed to evaluate differences in opioid prescribing practices among providers treating SCD in the US and internationally. A survey was disseminated electronically to known providers using a combination of purposive and snowball sampling strategy. There were 127 responses and 17 countries represented. US providers were more likely to prescribe opioids (*p* < 0.001) and were more likely to be “very comfortable” prescribing opioids than non-US prescribers (*p* < 0.001). US providers also tended to prescribe more tablets per patient of stronger opioids than non-US physicians. US physicians were more likely to be concerned that patients were abusing opioids than non-US physicians (32% vs. 27%, *p* < 0.05). There are significant variations in how different parts of the world manage pain in the outpatient setting for SCD. Identifying optimal home pain management strategies is necessary to improve care and long-term outcomes in SCD.

## 1. Introduction

The most common presentation for individuals with sickle cell disease (SCD) is acute painful episodes (vaso-occlusive crises (VOC)) [1,2], and these are the leading cause for hospital utilization and admission [3]. Estimated costs of health care for individuals in the US with SCD range from 1.1 billion [3] to 2.4 [4] billion. In an effort to decrease health care utilization, most individuals are prescribed oral analgesic medications for pain management at home [5]. These medications are highly variable but usually include anti-inflammatory drugs, muscle relaxants, anti-depressants and opioid analgesics.

For individuals with SCD presenting for acute care due to VOC, the 2014 National Heart, Lung and Blood Institute (NHLBI) guidelines [6] and the United Kingdom guidelines [7] both recommend rapid and aggressive management with parenteral opioids. These recommendations are supported by the patient population [8] who site acute pain as an emergency, requiring aggressive and sometimes high-dose opioid therapy, which leads to concerns of abuse by providers not used to caring for this population as well as inappropriate stigmatization [8,9,10]. Additionally, studies have shown that patients with acute VOC are more likely to improve more rapidly when parenteral pain medication is given in the first 60 min [6,7,11] of presentation, often allowing patients to have decreased total emergency department length of stay [12].

There have been studies attempting to characterize pain and medication use at home in the SCD population. The Pain in Sickle Cell Epidemiology Study (PiSCES) project conducted on adults living in the United States found that the majority of VOCs are managed at home, and that patients used opioids on 78% of their home pain days [13]. Even when pain episodes were considered severe, fewer than five percent of days were spent in hospitals or the Emergency Department [14]. In a large study describing pain management in adults with SCD, researchers in the Multicenter Study of Hydroxyurea found that at-home analgesics were used on 40% of diary days, with oxycodone and codeine used most often [15]. Despite these well executed studies, the optimal method of home-pain management has not been evaluated. Specifically, there are no large-scale clinical trials comparing the efficacy of different oral pain medications for home use and no established guidelines on home pain management regimens in SCD. 

Consequently, analgesic prescribing habits vary widely between providers and institutions, both domestically and internationally. The Determining Effects on Platelet Inhibition on Vaso-Occlusive Events (DOVE) study [16] was the first prospective, multinational study conducted in children with SCD and results found that opioid use varied significantly among regions but was the highest in the Americas compared to other regions [17].

Due to the paucity of available data on optimal analgesic medication for home pain in SCD as well as a lack of comparative data, this study aimed to describe the opioid prescribing habits among international providers treating individuals with SCD. The goal of this study was to begin to assess variations in medications used for outpatient and inpatient management of SCD-related pain between prescribers in different countries in order to plan a prospective comparative study of pain management. 

## 2. Materials and Methods

This study was Institutional Review Board approved at the Medical University of South Carolina. A thirty-question survey was created using Redcap version 8.6.5 and sent out electronically with a brief description of the research and the purpose of this study (Appendix A). Most of the survey focused on outpatient and inpatient pain management, with additional questions regarding medication availability and use, including hydroxyurea. Providers were recruited using a combination of purposive and snowball sampling strategy starting with databases of providers who have attended SCD-specific conferences. Initial survey participants were selected based on their attendance at previous hemoglobinopathy-specific meetings or participation in sickle cell disease specific projects. Recipients of these initial 150 emails were asked to refer other providers treating and managing individuals with SCD. The survey was closed when <1 response per day was received for 5 consecutive days. Respondents were not asked to provide information about their specific hospital/center of practice or their specific practice size. They were asked general questions at to the size of their practice. The survey included both closed and open-ended questions. Responses to questions were anonymous, unless the participant elected to provide their email address to obtain the results of the survey afterwards. Responses were compared between US and non-US providers using chi square tests and Fisher’s exact tests. We also used multivariable logistic regression models to examine the associations between provider location (US vs. non-US) and survey responses, while adjusting for several covariates, including provider type (i.e., primarily pediatric provider vs. other) and numbers of adults and children with SCD treated at the provider’s center. All analyses were conducted using SAS version 9.4 (SAS Institute, Cary, NC, United States.

## 3. Results

A total of 127 responses to the survey were received. The survey remained open for six weeks. Because of the snowball sampling methodology utilized in the project, the actual response rate was not estimable. 

There were 17 countries represented in the sample. There were multiple responders from the US (59%) as well as a substantial international response. Most of the respondents were hematologists/oncologists, with representation from both pediatric (43%) and adult (34%) providers (Table 1). Other physician provider groups included internal medicine (*n* = 4), emergency department (*n* = 9), family medicine (*n* = 3), and other unspecified disciplines (*n* = 13) (Table 1).

### 3.1. Outpatient

The survey included questions regarding types of pain medication prescribed, medication dosing and quantity and whether protocol or individual-based dosing strategies were used. When prescribing outpatient pain management, most providers did not use a universal (or protocol-based) dosing regimen or strategy. Instead, 90% of providers reported that they used an individualized dosing regimen (and did not prescribe the same opioid medications for every patient). Overall, providers from the United States (US) were more likely to prescribe opioids for home pain management than non-US physicians (95% vs. 73%, *p* < 0.001) (Table 2) and were more likely to be “very comfortable” prescribing opioids than non-US physicians (73% vs. 37%, *p* < 0.001). Of those physicians that do not prescribe opioids for outpatient management of pain, non-US physicians were more likely to attribute it to outpatient opioids not being standard of care or not being “allowed” (50% vs. 25%), although this was not statistically significant.

### 3.2. Outpatient Quantity

Of those physicians who prescribe opioids, most (70%) prescribed 30 doses or less at a time. However, non-US physicians were more likely to prescribe less than 10 doses at a time (64% vs. 17%, *p* < 0.001) (Table 2). 

### 3.3. Outpatient Pain Medication Type

**Figure 1:**
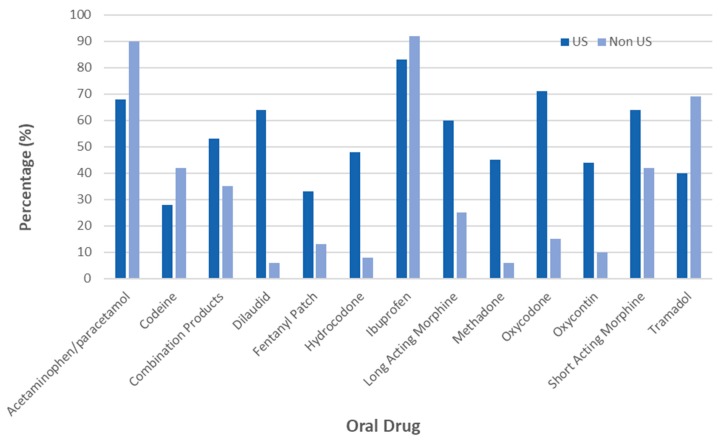
Oral analgesia typically prescribed for patients with sickle cell disease in the United States and internationally. Physicians in the United States prescribed stronger opioids more often, including dilaudid, long-acting morphine, methadone, hydrocodone, fentanyl patch oxycodone, oxycontin and short-acting morphine than non-US countries. Non-US counties used tramadol, ibuprofen and acetaminophen/paracetamol more often than the US.

Overall, the top five most commonly prescribed medications for home pain management were acetaminophen/paracetamol (98%), ibuprofen (88%), short-acting morphine (55%), tramadol (52%) and oxycodone (48%) (Figure 1). Non-US physicians were more likely to prescribe non-opioid medication as first line acetaminophen/paracetamol (90% vs. 68%, *p* < 0.05) and lower-potency opioid, tramadol (69% vs. 30%, *p* < 0.05) if necessary. Non-US physicians rarely prescribed any long-acting opioid medication. In contrast, US providers were more likely to prescribe short-acting morphine (64% vs. 42%, *p* < 0.05), long-acting morphine (60% vs. 25%, *p* < 0.001), oxycodone (70% vs. 15%, *p* < 0.001), oxycontin (44% vs. 10%, *p* < 0.001) and hydromorphone (64% vs. 6%, *p* < 0.001) (Figure 1). 

### 3.4. Inpatient

**Figure 2:**
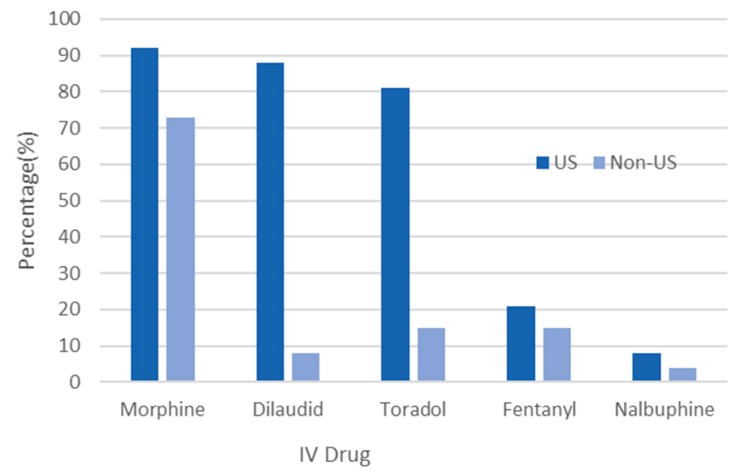
Intravenous analgesia for inpatient use in patients with sickle cell disease in the United States and internationally. For intravenous (IV) pain use, physicians from the United Sates used all listed opioids more often than non-US countries. IV dilaudid and toradol were used almost exclusively in the United States.

The survey also asked questions regarding inpatient pain management for SCD. This included information on the type of pain medication used for acute sickle cell pain or VOC. The most commonly prescribed acute care parenteral medications were morphine (84%), hydromorphone (55%), and toradol (54%) (Figure 2). Of interest, intravenous hydromorphone (dilaudid) was used almost entirely in the US alone (88% vs. 8%, *p* < 0.001).

### 3.5. Opioid Misuse/Abuse

Physicians in the US were more likely to be concerned that patients were abusing opioids than non-US physicians (32% vs. 27%), although this finding was not statistically significant in the multivariable analyses and were more likely to perform routine urine drug screens (42% vs. 4%, *p* < 0.001). Providers in the US were also more likely to use a prescribing database to track prescriptions than non-US physicians (78% vs. 36%, *p* < 0.0001) (Table 1). Interestingly, if providers were concerned about opioid abuse or misuse, non-US physicians were more likely to mandate counseling than US physicians (56% vs. 36%, *p* < 0.05).

### 3.6. Disease-Modifying Therapy

In addition to questions of pain management, the survey also included questions on other SCD-specific management therapies. These included questions about hydroxyurea use and management and chronic transfusions as a proxy to assess both the providers comfort with treating individuals with SCD and to evaluate access to medication (overall). The large majority (92%) of providers surveyed prescribed hydroxyurea, suggesting adequate access to the medication in all countries evaluated (Table 3). However, there was a greater percentage of providers prescribing hydroxyurea therapy in other countries than in the US (Table 3). There was no significant difference in the percentages of patients on chronic transfusions in the US and other countries (Table 3).

### 3.7. Pediatric vs. Adult Providers

Investigating whether providers who primarily treat children with SCD exhibit different opioid prescribing patterns than providers who primarily treat adults with SCD was beyond the scope of this study. Nevertheless, it is interesting to note than in our multivariable statistical models, there were some differences between provider types (i.e., pediatrics vs. adults) that were statistically significant. Specifically, the proportion who responded that they were somewhat or very comfortable in prescribing opioids was higher among pediatric providers (100.0% vs. 89.4%, *p* < 0.05), and the proportion who responded that over 50% of their SCD patients are on hydroxyurea was higher among pediatric providers (63.2% vs. 36.4%, *p* < 0.001). 

## 4. Discussion

Pain, usually caused by acute vaso-occlusive episodes, is the most common reason for health care utilization in SCD. Current guidelines indicate that IV opioids are the recommended treatment for these crises in the acute care setting [6]. However, affected individuals are often encouraged -and attempt to- manage their pain at home rather than coming to the emergency department/hospital. Thus, it is important to evaluate current pain management strategies in different countries to eventually understand how these strategies relate to overall disease management, quality of life and overall outcomes including hospital utilization. Furthermore, there is an increased number of novel therapeutics in development for SCD. As many of the clinical trials are now multinational and utilize measure of pain or acute care as an endpoint, it is important to assess differences in pain management across different countries. 

This study demonstrated that although providers in many countries use oral opioids for the outpatient management of SCD-related pain, providers in the US were more likely to prescribe stronger opioids and more doses of opioids for outpatient management. When providers in other countries did prescribe outpatient medications for pain, it was more likely to be acetaminophen or ibuprofen first, followed by less potent opioids like tramadol. This difference in prescribing habits could be due to more availability of opioids in the US as evidenced by the fact that the US is one of the nations that consumes the highest percentage of the world’s opioids [18] or because US physicians were more comfortable prescribing opioids. However, this difference in practice is very important to explore as it relates to overall outcomes in SCD, frequency of acute care utilization, and quality of life for affected individuals.

It is interesting to note that the differences seen in prescribing habits are defined mostly by the geographic location of the provider (US. vs. Non-US) as opposed to the age of the patients for whom the provider is prescribing. Specifically, it would seem more likely that more opioids would be prescribed for adults due to the cumulative damage caused by SCD and the development of chronic pain. However, as noted in Table 2, the differences noted between US vs. non-US physicians were similar between physicians who primarily treat pediatric patients and physicians who do not focus primarily on pediatric patients. Thus, while we would expect that opioid prescribing would change significantly from pediatric to adult care, the differences noted in this study were far more due to the geographic location of the provider. There is also a significant difference in hydroxyurea prescribing in non-US countries compared to US countries as noted in Table 3. It is unclear whether the decrease in the use of hydroxyurea contributes to the increase in the prescribing of opioids. Clearly, this finding also requires further study. 

These age-related findings are of specific importance, since there are several potential complications for long-term opioid use. One of these concerns is the development of opioid-induced hyperalgesia (OIH). This complication of opioid pain medications causes increased sensitivity to pain with increasing exposure to opioids [19]. Long-term, OIH can lead to increase in hospital utilization and decrease in quality of life [20]. Another well-described complication from chronic opioid use is bowel dysmotility or “opioid bowel.” As with OIH, this secondary problem results in increased pain for the affected individual as well as potentially more life-threating results such as bowel obstruction [21]. Overall, there is evidence to support chronic opioid (in cancer pain) use. However, long-term safety and efficacy of prolonged opioid use has not been established [22]. As more individuals with SCD are living longer, more research is needed on outpatient pain management and the effect of different regimens on outcomes, including the development of chronic pain, secondary complications, and quality of life. This need is further highlighted by the significant geographic variability in medication prescribing habits in the both the inpatient and outpatient settings as demonstrated in this study, without sufficient comparative studies to determine optimal practice. Additional studies are also needed to compare quality of life, functional outcomes and overall complication rate in patients with SCD-related chronic pain in these different geographic areas.

Compounding the lack of objective criteria on medication regimens for home pain management is the concern for opioid abuse and misuse. There is currently a global concern for increased opioid dependence and abuse. The World Health Organization estimated that 27 million people suffered from opioid use disorders in 2016 [23]. In the United States, an estimated 11.5 million people misused prescription opioids [24]. In Europe there has been an increased demand for treatment related to prescription opioids, and in Africa and Asia there has been growing concerns for misuse of tramadol [19].

There is no direct evidence that individuals with SCD sickle cell patients experience higher rates of opioid abuse and dependence than the general population. In fact, a recent publication by Ballas and colleagues demonstrate that the increase in opioid-related deaths is not attributable to individuals with SCD. However, ongoing concerns remain with prescribers [25].

Contrary to this evidence, survey respondents in the US did express more concern about opioid misuse and abuse and were more likely to perform routine urine drug screens and to use prescribing databases to track prescriptions than non-US physicians. However, it is possible that without objective criteria to guide prescribing, some providers may overestimate the prevalence of opioid abuse or dependence [26]. Additionally, fear of punishment or repercussions from drug-enforcement agencies for not assessing individual patient use may also drive current practice.

There were some noted limitations to this study. The initial survey targeted providers who had previously attended SCD-specific meetings/conferences and were more likely to be part of SCD centers. Snowball sampling allowed for additional respondents in other areas, but the majority of responses were likely received from individuals with more expertise in SCD (as also evidenced by the amount of disease-modifying therapy prescribed by these providers). Thus, this study is not entirely representative of all of the providers treating individuals living with SCD. However, the methodology provides a strong foundation by which to understand practices in different geographic locations regardless of the level of SCD specialization of the providers. Further, the authors are aware that medication availability differs between US and non-US countries. This study was undertaken in part, to highlight these differences which are important in considering comparative therapies in different countries. This study does not speak to which medications or pain medications providers would prefer to use. Instead, it is asking what the current practice of these providers in these countries are, which may differ based on medication availability. The data does support that some non-US countries are restricted from providing opioids, which supports the premise that these differing methods of treatment should be further analyzed. 

Our study supports the need for future, international research to study optimal strategies for home management of vaso-occlusive crises and chronic pain in individuals with SCD. Given the wide geographic differences in prescribing habits, there is a significant need for safe and efficacious multinational guidelines for the prescribing of oral opioids. These findings also highlight the importance of considering these geographic differences when pursuing multi-national clinical trials. Furthermore, differences in pain management (both inpatient and outpatient) also highlight the need to identify disease-specific endpoints not related to pain or acute care use. Lastly, alternative disease-modifying agents for the prevention of SCD-related pain remain a huge unmet need and may reduce the need for opioid use at home in the future.

## Figures and Tables

**Table 1:** Demographics and use of sickle cell disease (SCD) therapies by survey respondents (*n* = 127).

Type of Physician	*N* (%)
Pediatric Hematologist/Oncologist	54 (42.5%)
Adult Hematologist/Oncologist	44 (34.7%)
Internal Medicine	4 (3.2%)
ER Physician	9 (7.1%)
Family Medicine	3 (2.4%)
Other	13 (10.2%)
**Regions**	***N* (%)**
United States	75 (59)
Europe	25 (19.7%)
Africa	12 (9.4%)
South America	7 (5.5%)
Caribbean	3 (2.4%)
Other	5 (3.9%)

**Table 2:** Summarized responses to survey questions by United States versus non-United States providers, *n* = 127.

Question	Response	Peds *US *n* (%)	Peds non-US *n* (%)	Other US *n* (%)	Other Non-US *n* (%)	Total US *n* (%)	Total Non-US *n* (%)	US vs. Non-US Unadjusted *p*-Value	US vs. Non-US Adjusted † *p*-Value
**Do you prescribe opioids?**	Yes	35 (100.0)	17 (68.0)	35 (89.7)	21 (77.8)	70 (94.6)	38 (73.1)	*p* < 0.0007	*p* < 0.05
	No	0 (0.0)	8 (32.0)	4 (10.3)	6 (22.2)	4 (5.4)	14 (26.9)		
**How comfortable are you prescribing opioids?**	Very Comfortable	32 (91.4)	9 (36.0)	22 (56.4)	10 (37.0)	54 (73.0)	19 (36.5)	*p* < 0.0005	*p* < 0.0001
	Somewhat Comfortable	3 (8.6)	16 (64.0)	14 (35.9)	13 (48.2)	17 (23.0)	29 (55.8)		
	Somewhat Uncomfortable	0 (0.0)	0 (0.0)	1 (2.6)	3 (11.1)	1 (1.4)	3 (5.8)		
	Very Uncomfortable	0 (0.0)	0 (0.0)	2 (5.1)	1 (3.7)	2 (2.7)	1 (1.9)		
**How many doses do you prescribe at one time?**	<10	4 (11.8)	11 (57.9)	8 (23.5)	16 (69.6)	12 (17.7)	27 (64.3)	*p* < 0.0001	*p* < 0.0001
	10–30	19 (55.9)	8 (42.1)	8 (23.5)	5 (21.7)	27 (40.0)	13 (31.0)		
	30–60	10 (29.4)	0 (0.0)	8 (23.5)	1 (4.4)	18 (26.5)	1(2.4)		
	60–90	0 (0.0)	0 (0.0)	5 (14.7)	1 (4.4)	5 (7.4)	1 (2.4)		
	>90	1 (2.9)	0 (0.0)	5 (14.7)	0 (0.0)	6 (8.8)	0 (0.0)		
**Prescription tracker database?**	Yes	27 (77.1)	9 (36.0)	30 (79.0)	9 (36.0)	57 (78.0)	18 (36.0)	*p* < 0.0001	*p* < 0.0001
	No	8 (22.9)	16 (64.0)	8 (21.1)	16 (64.0)	16 (22.0)	32 (64.0)		
**How often are you concerned for misuse?**	Never	4 (11.4)	11 (44.0)	0 (0.0)	4 (14.8)	4 (5.4)	15 (28.9)	*p* < 0.0002	*p* < 0.05
	Not very often	30 (85.7)	12 (48.0)	29 (74.4)	17 (63.0)	59 (79.7)	29 (55.8)		
	Somewhat often	1 (2.9)	1 (4.0)	9 (23.1)	5 (18.5)	10 (13.5)	6 (11.5)		
	Very Often	0 (0.0)	1 (4.0)	1 (2.6)	1 (3.7)	1 (1.4)	2 (3.9)		
**How often are you concerned for abuse?**	Never	1 (2.9)	5 (20.8)	0 (0.0)	1 (3.7)	1 (1.4)	6 (11.8)	*p* < 0.05	*p* = 0.15
	Not very often	29 (82.9)	15 (62.5)	20 (51.3)	16 (59.3)	49 (66.2)	31 (60.8)		
	Somewhat often	4 (11.4)	2 (8.3)	15 (38.5)	6 (22.2)	19 (25.7)	8 (15.7)		
	Very Often	1 (2.9)	2 (8.3)	4 (10.3)	4 (14.8)	5 (6.8)	6 (11.8)		
**Do you do urine drug screens?**	Yes	7 (21.2)	1 (4.4)	23 (60.5)	1 (3.7)	30 (42.2)	2 (4.0)	*p* < 0.0001	*p* < 0.0001

* Pediatrics, **^†^** Using multivariable logistic regression models, the *p*-values were adjusted for whether the provider primarily cared for adult vs. pediatric patients, the number of children with SCD treated by their center, and the number of adults with SCD treated at their center.

**Table 3:** Use of disease-modifying therapy in the US and other countries, *n* = 127.

THERAPY	Total *N* (%)	Peds US *N* (%)	Peds Non-US *N* (%)	Other * US *N* (%)	Other * Non-US *N* (%)	Total US *N* (%)	Total Non-US *N* (%)	US vs. Non-US Unadjusted *p*-Value	US vs. Non-US Adjusted ^†^ *p*-Value
**Providers that Prescribe Hydroxyurea**									
Yes	115 (92.7)	35 (100.0)	25 (100.0)	29 (76.3)	26 (100.0)	64 (87.7)	51 (100.0)	*p* < 0.01	*p* < 0.01
**Estimated Percentage of Patients Taking Hydroxyurea**									
<10%	8 (7.1)	0 (0.0)	1 (4.2)	1 (3.5)	6 (23.1)	1 (1.6)	7 (14.0)	*p* < 0.01	*p* < 0.01
10–30%	20 (17.9)	5 (15.2)	4 (16.7)	1 (3.5)	10 (38.5)	6 (9.7)	14 (28.0)		
30–50%	28 (25)	5 (15.2)	6 (25.0)	13 (44.8)	4 (15.4)	18 (29.0)	10 (20.0)		
50–80%	40 (35.7)	17 (51.5)	5 (20.8)	12 (41.4)	6 (23.1)	29 (46.8)	11 (22.0)		
80–100%	16 (14.3)	6 (18.2)	8 (33.3)	2 (6.9)	0 (0.0)	8 (13.0)	8 (16.0)		
**On Chronic Transfusions**									
<10%	68 (54)	17 (48.6)	16 (64.0)	21 (53.9)	14 (51.9)	31 (51.4)	30 (57.7)	*p* = 0.19	*p* = 0.63
10–30%	51 (40.5)	18 (51.4)	5 (20.0)	16 (41.0)	12 (44.4)	34 (46.0)	17 (32.7)		
30–50%	5 (4)	0 (0.0)	3 (12.0)	1 (2.6)	1 (3.7)	1 (1.4)	4 (7.7)		
50–80%	2 (1.6)	0 (0.0)	1 (4.0)	1 (2.6)	0 (0.0)	1 (1.4)	1 (1.9)		

* Other refers to providers who are not primarily pediatric providers. **^†^** Using multivariable logistic regression models, the p-values were adjusted for whether the provider primarily cared for adult vs. pediatric patients, the number of children with SCD treated by their center, and the number of adults with SCD treated at their center.

## Appendix A

Thirty question survey created for this study.
